# What Is Meant by Insanity

**Published:** 1911-02-18

**Authors:** Maurice Craig

**Affiliations:** Lecturer on Mental Diseases at Guy's Hospital Medical School.


### Hospital Clinics.

### WHAT IS MEANT BY INSANITY.

By MAURICE CRAIG, M.D., F.R.C.P., Lecturer on Mental Diseases at Guy's Hospital Medical School.

(A Clinical Lecture delivered at Guy's Hospital.)*

As is my usual custom before starting these lectures I must ask you to banish from your minds all preconceived ideas as to what is meant by insanity. Never use the words "madness," "lunatic," or such obsolete terms, as they convey an entirely different meaning from what I hope to be able to show you is the real meaning of mental disorder. Approach the study of mental disorder in the same way as you would approach any other branch of medicine, for there is really no distinction between mental disorder and physical disorder. The distinction which has been made in the past is purely an arbitrary one, and one which no doubt has its basis upon superstition. It is as useless for me to define insanity as it would be to define what one means by soundness of mind. Sanity itself is a relative term, and is equally indefinable. Every physician knows what he himself means when he speaks of a normal or healthy body, but would he care to state such an opinion in the terms of a definition? A body may have many blemishes and yet be classed as healthy, for experience enables us to distinguish between those defects which are important and those which are immaterial. So it is with the mind. There is no such thing as perfection in mind or body. Experience alone can furnish us with a knowledge by which we can form a judgment of what, for want of a better term, may be called a "normal standard."

Clearly, then, we ought to understand something about the mind itself before we attempt to gauge the various disorders by which it may be disturbed. I do not wish to worry you with problems of psychology, but throughout these lectures I must briefly touch upon various attributes of normal mind, and point out to you how and why they may become disordered. I shall attempt to instil into you the principles of mental disorder, explaining to you the various symptoms that may arise, and at Bethlem these symptoms will be grouped together in the demonstrations illustrating the various types of insanity as known at the present day.

Now in studying mental disorders there are three main factors which have to be considered:—

1. Self, which is the sum total of sensations, perceptions, feelings, and ideas of any given moment.
2. The environment or surrounding in which we live.
3. The adjustment of the one to the other.

To deal with these:—

1. As I shall show you in a subsequent lecture, in the early months of life we have no idea of personality, and even when the child begins to talk it speaks of itself in the third person; but the normal infant is constantly receiving sensations by which it will ultimately know its relationship to the world. Tactual sensation is the one by which we probably learn the most. A child at first stretches out for objects far beyond its reach, but slowly acquires the knowledge of spacial perception. A child learns a language by which it acquires the knowledge of the thoughts of others. In fact, to put it shortly, a child is continually acquiring, by means of special sense and organic sensations, its future knowledge of self. At the same time, these sensations are being associated with feeling-tones of pleasantness and unpleasantness. Sensations are being stored up as memory ideas, to be recalled again as occasion demands. Every time a movement is made we receive a group of sensorial impressions occasioned by and peculiar to that movement. Diminish sensation and you have, to a certain extent, taken away the consciousness of self. Sensation, then, is probably the basis of the knowledge of self, and, as I shall endeavour to show to you, disorders of sensation give rise to disorders of this knowledge of self. In other words, altered sensation means altered personality. The ego, composed as it is of the sum total of subjective sensations, etc., is continually changing; the ego of one moment is not the ego of the next. Compare yourself, for instance, in health or in pain, when rested or when fatigued, and you will appreciate what I mean by the effect of changed sensations on personality.

2. By environment we mean the different grades of society which we meet and the various circumstances by which we are surrounded, and, as you will appreciate, this, like sensation, is an ever-changing quality.

3. Now it is the adjustment of these two factors which is the very essence of conduct; and when one appreciates that each of them, as I tell you, is a varying quantity, you will recognise how difficult this adjustment is, and any failure of it will tend to place the person out of touch with the community in which he lives.

In insanity we have to deal with this failure of adjustment of self to environment. The patient may neglect the most necessary and rudimentary requirements of life: food may neither be sought nor eaten even when placed within reach; the ordinary laws of self-conservation may be neglected; rules of personal cleanliness may be unobserved; ability to earn a living may be absent; acts of violence against self and others may be committed; we may be unable to distinguish between what belongs to us and to others; in these and in many other ways the disorder of conduct may show itself.

Society rules that the liberty of the subject is only possible so long as that liberty is not used to interfere with the liberty of others.

Now, for a moment, let us just consider how

* We are indebted to the Editor of the *Guy's Hospital Gazette* for a report of this lecture.—Ed. The Hospital.

altered sensation may lead to altered personality, and altered personality in turn may seriously affect our conduct. To begin with the lesser changes: let us suppose that the head, limbs, and chest of a patient are anæsthetic. This will give rise to a comparative hyperæsthesia of the abdominal area, and the result will be very similar to what it would be were the abdominal area hyperæsthetic as compared with a normal state of the other parts of the body. This hyperæsthesia may give rise to disagreeable sensations in the stomach and intestinal tract. In other words, this individual's personality will be largely coloured by the sensations he is deriving from these special areas of the body. The next stage is for this individual to account for the abnormal sensations that he is receiving, and in so doing an erroneous judgment may be formed, and this we speak of as a delusion. And let me tell you at once that a delusion is a false belief, but the question as to whether the person is of unsound mind must be decided apart from the delusions, for clearly a sane person may have a false belief. To the physician the importance, then, is not the delusion, but why the delusion is there. Delusions may bias conduct, and, what is equally important, they form a basis by which a person will collect evidence to favour his ideas and ignore the weight of evidence against them. So again with special sensory disturbances, the patient may hear sounds which those about do not hear, or see objects unseen by others, and although such hallucinations are certainly the product of some abnormal working of the brain, they are frequently found in persons who are perfectly sane in that they are capable of doing their daily work and in every way conforming to the social laws. Therefore, either delusions or hallucinations standing alone and unsupported by other evidence are not conclusive of insanity. But if they co-exist—that is, that the man not only has a false belief, but that this false belief is supported by auditory or visual hallucinations—clearly this is stronger evidence of unsoundness of mind. Further, if in addition there are serious disorders of conduct, then the case is probably one of certifiable insanity. Medical experts, when acting as witnesses in our courts of justice, frequently have their statements in this way dissected by a skilful counsel. They are asked in threatening tones whether they wish to tell the jury that hallucinations are indicative of insanity, that delusions only exist in the minds of the insane, and, maybe, that emotional disturbance, such as depression of spirits, connotes insanity? To each question the witness must answer "No," but let him seize his opportunity to add that when associated together there is strong presumptive evidence of serious mental disorder. Mental is in every way comparable to physical disorder. It is only by the grouping of symptoms that we are able to diagnose various diseases; so it is only by a collection of data that we are able to deduce insanity.

As I shall endeavour to show you, emotions play an important part in the social life of the individual. I have already spoken of "feeling tones," by which one means the pleasantness or unpleasantness associated with simple sensations. But emotions are more complex; the pleasantness and unpleasantness are associated with organic sensations, and changes take place in the secretions; for example, the surface of the body may be bathed in perspiration, the mouth may be dry, or the eyes wet with tears. In health there should be perfect harmony between the emotions and other mental processes, but changes may take place in which the emotions become disordered, and one feeling tone may pass into the ascendant, with the result that the mental life of the individual for the time being is under the influence of this colouring, be it one of gloom or of uncontrolled buoyancy. But here again we come back upon sensation, because emotion is associated with sensations, or former sensations, and is merely the affective side of these sensations. Depression of spirits is normal under certain conditions, but the misery may pass beyond all bounds, and no longer merely colour the original ideas with which it was associated, but colour every action and thought of that individual's life. It is only human nature to endeavour to explain all our sensations and feelings, and it is in explaining disordered sensations and feelings that the maniacal or melancholic patient forms erroneous conclusions; in other words, the former develops what we choose to call "delusions" of exaltation, and the latter "delusions" that he is lost or ruined.

Attention, again, is an attribute of mind, and in health we ought to be what is called polyideational, that is, we should be capable of concentration on any subject which at the moment demands our attention. On the other hand, we may become inattentive. Concentration upon a subject may become impossible, and we may pass into a condition closely resembling a dream state, a condition in which extreme inattention is one of the chief characteristics. Or the disorder of attention may be that of hyperattention, a state in which one idea is constantly asserting itself into consciousness. And perhaps this is one of the best illustrations I can give you of what one means by unsoundness of mind, and how near it is to health, and yet how close it may be to a state of certifiable insanity. An obsession is a morbid impression or idea imperiously demanding notice; in other words, one thought is constantly forcing itself upon us. Turning off the gas at night is a common type of obsession. As soon as a man has put out the light the thought occurs to him that he may not have properly turned off the tap, and he returns to inspect it. So far there is nothing abnormal, as he does nothing more than a conscientious person might do. But suppose that he no sooner reaches his bedroom than he begins to wonder whether in removing his hand after turning off the light he just touched the tap again, and may have caused an escape. For a time he may resist the impulse to go down and inspect the gas-bracket, but gradually the idea becomes more and more reinforced, as he thinks of the consequences that may arise, until he ultimately gives way. You may consider that, so far, all the man has done is to doubt the fine adjustment of his fingers in the performance of a movement. He is once more back in his room. when the old idea again haunts him. He recognises the folly of it, and refuses to obey its dictates. He tries to think of other things, but his attention always reverts to the one thought, and in desperation he again returns to inspect the gaspipe. With each recurrence the mental state becomes one of greater agitation. Not only is he disturbed at the repetition of the thought, but his powerlessness to disregard it. And you can see that if the obsession recurs frequently enough, if it occasions disturbance of his sleep, if, in other words, he is spending the night in a state of mental agitation, his conduct has by now become so disordered that he may have passed within the realm of certifiable insanity. Also other symptoms may have developed; active depression, with all its concomitant symptoms, especially that most trying one of all, the feeling that life is not worth living, which may at any time end in an attempt at self-destruction.

Memory, again, is an important attribute of the mind. The healthy brain should be able to store fresh impressions, and new facts should become associated with former ideas. Loss of memory may seriously affect conduct, and an amnesic person may seriously contravene moral and social codes; or it may lead to such disorientation that he is unable to know his whereabouts.

Self-Analysis as an Aid to the Study of Insanity.

I think I have now given you sufficient illustration to prove to you how altered sensation may lead to altered feelings, and how each or both may so affect our personality that our outlook upon life may become entirely changed. If you wish to understand mental disorder and how a person feels, you may study it yourself in the different phases of your mental life. Analyse your feelings on your most gloomy days and try to imagine yourself ten times more miserable, and then you will have some idea of what depression really is. When you are worried you are unable to settle to anything; you pick up a book and try to read, and soon close it, as you cannot fix your attention upon the subject. Imagine yourself always in this state and you will have a faint idea of real mental disorder. As a child you may have experienced the feeling that someone was following you as you hurried along some dark lane or passage; imagine yourself always in this state and you will have a slight conception of what it is to be haunted by the thought that you are being continually followed. Go by underground railway to some station that you have never been to before, and as you stand outside the station you look round and the sensations which you receive give you no feeling of familiarity, and you become totally disorientated, not knowing your whereabouts or which way to turn to reach the place you seek. Such temporary disorientation will give you some slight insight into the state of a man who has more permanently lost his memory; in other words, his knowledge of people and things.

If you endeavour to analyse mental disorder in this way, the subject will at least become intelligible, and will no longer appear to you to be a mere concatenation of strange symptoms. The error that most persons make is, that they fail to realise that insanity consists largely of exaggerations of those mental aberrations to which the majority of individuals are liable.

To sum up, mental unsoundness may be either due to failure of evolution or a result of dissolution. The former is usually classed under the headings Idiocy, Imbecility or Congenital Mental Enfeeblement. Now, by failure of evolution all we mean is, that the attributes of mind which should have developed during the early years of a child's life have not done so, and that the child reaches maturity lacking in control, devoid of moral sense, with no power of attention, or is in some other way defective in its mental equipment. On the other hand, just as we have acquired various attributes of mind (largely, as I have shown you, by means of our special sense sensations), so we may again lose them owing to disease. When we do lose them, we do so in the inverse order to that in which we attained them, the latest acquirements being the first affected. Even with fatigue, we have lessened power of attention, diminished power of association, which, in turn, affects our memory. Our powers of control are lessened, as exhibited by outbursts of irritability, and our judgment becomes inaccurate and unreliable. Loss of control is shown again by restlessness or fidgety movements, which closely resemble those observed in children. The weary man of business paces up and down his office trying to concentrate his thoughts, and the more exhausted he is the more energetic he becomes.

Fatigue Symptoms.

Many of the early changes are similar to those of fatigue conditions, and they become progressively more marked, especially if, as is usual, the importance of them is not recognised, or they remain untreated. As the condition advances, the various systems of the body become affected, and insomnia may hasten the collapse. Whilst all this is taking place, the conduct begins to change, and becomes more and more uncertain. It is then that the question arises whether the day has not come when the patient should be placed under care. When we come to discuss treatment, I shall impress upon you the importance of recognising these early changes, for the longer one works at disorders of mind, the more convinced one becomes that the more serious disturbances are due to the early symptoms having been overlooked or neglected. Disordered sensation or strong emotion may usurp the whole attention, sensory illusion may deceive the man, or profound depression may paralyse both thought and movement. But, let it be remembered, insanity is not revealed by one symptom; the change can be seen in everything, physical or mental. In determining insanity the evidence to establish it cannot be decided from one symptom. The symptom present may be regarded much in the same way as pieces of circumstantial evidence are during a trial. Each individual piece may denote nothing, but the chain formed by welding the separate pieces together may be so strong as to compel one conclusion. So with the symptoms of insanity; each of them if present alone might be consistent with sanity, but taken together they may form so strong a body of evidence as to force the inference of insanity.

